# Laryngopharyngeal reflux induced sleep-related laryngospasm

**DOI:** 10.1007/s11845-022-02934-x

**Published:** 2022-01-31

**Authors:** Ross O’Shea, Máire Gaffney, Majura Kaare, John Eugene Fenton

**Affiliations:** 1 Faculty of Education and Health Sciences, University of Limerick Medical School, Garraun, Castletroy Limerick, Ireland; 2 Department of Otorhinolaryngology Head and Neck Surgery, University Limerick Medical School, Limerick, Ireland

**Keywords:** Diagnosis < General, Gastro-oesophageal reflux < Pharynx, Larynx, Obstructive sleep apnoea < Rhinology, Sleep medicine < General, Stridor < Larynx

## Abstract

**Background:**

Sleep-related laryngospasm (SRL) has been defined as the sustained closure of the vocal cords during sleep. Studies have suggested that it is a rare manifestation of laryngopharyngeal reflux (LPR). Difficulties in diagnosing SRL and LPR have led to the condition being under-recognised in the clinical setting.

**Aims:**

The aim of this study was to determine if LPR was the cause of the SRL symptoms seen in our patients.

**Methods:**

A retrospective chart assessment of patients with SRL. Patients with risk factors for LPR were identified. These included smoking status, alcohol intake, a history of dyspepsia or history of gastroesophageal reflux disease, a history of late-night eating and a history of eating spicy or fatty foods before bed. A clinical diagnosis based on the history and response to management was made for the diagnosis of LPR. All were advised to refrain from late meals and those with signs of nasopharyngitis were commenced on proton pump inhibitor therapy.

**Results:**

Nineteen patients (mean age ± SD: 57.21 ± 15.18) were included in the study. All had at least one risk factor for LPR. Ten (52.6%) had signs of nasopharyngitis on nasendoscopy. Following treatment, 17 (89.5%) reported no further SRL symptoms at 1-year follow-up.

**Conclusion:**

SRL is a largely unknown and under-diagnosed condition. We believe this study provides supportive evidence for the causal relationship between LPR and SRL.

## Introduction

Laryngospasm has been defined as the sustained closure of the vocal cords which results in partial or complete loss of airway patency [1]. Sleep-related laryngospasm (SRL) exclusively occurs while the patient is asleep and is characterised by sudden awakening with an inability to breathe. These episodes last seconds and can also be associated with stridor [2]. Patients present clinically well. They report episodes of acute choking attacks that occur at night and are short lasting. Clinical features and polysomnographic studies have previously suggested SRL to be a spasm of the vocal cords of unknown aetiology [3]. Fear of suffocation while sleeping has also been reported which has a detrimental effect on the patient’s quality of life [2]. Despite this, SRL is a condition not well described in the literature.

The term “sleep-related laryngospasm” was first described in a case series by Aloe and Thorpy which suggested laryngopharyngeal reflux (LPR) was a possible explanation for SRL [3]. LPR refers to the acute or chronic backflow of gastric contents of liquid or aerosol, into the larynx and/or pharynx and is considered to be relatively common in the general population. Although termed “silent reflux” in the past, it can present with a multitude of complaints involving the upper aerodigestive tract including an association with globus pharyngeus, chronic rhinosinusitis, a sore throat, dysphagia, chronic cough, and hoarseness [4]. SRL is believed to be a rare but severe manifestation of LPR [2, 5]. Lower oesophageal sphincter dysfunction is likely to be the probable cause of LPR. Risk factors for LPR include late meals, spicy or fatty foods, stress, cigarette smoking, caffeine intake and alcohol consumption [4, 6]. The incidence of SRL is still unknown but previous studies have estimated that 10–30% of patients that seek ENT care, present with symptoms of LPR [7].

Differential diagnoses for SRL include obstructive sleep apnoea (OSA), nocturnal asthma, sleep terror episodes and seizures. These need to be excluded in order for an accurate diagnosis of SRL to be made. OSA is the most common sleep-related breathing disorder and therefore must be ruled out before diagnosing SRL. Patients with OSA will not generally awaken themselves and will not report stridor nor difficulty breathing when awake. If such symptoms are present, then a likely diagnosis of SRL can be made; however, if the history is suggestive of OSA or nocturnal asthma, then the patient should be referred for a respiratory assessment.

LPR is notoriously difficult to diagnose and remains a controversial condition. Definitive criteria have not been universally agreed although multiple suggestions have been proffered. Diagnosis and management vary significantly from country to country [8]. LPR scoring systems based on the history and findings include the reflux finding score (RFS), reflux symptom index (RSI), reflux sign assessment (RSA), Horvath score and Sataloff score, which are cumbersome to perform in a busy clinic [7–10]. None of these scoring systems have included SRL as a factor. A previous study has demonstrated a relationship between sleep-related gastroesophageal reflux and SRL through the use of overnight polysomnogram and pH monitoring; however, these investigations are not readily available in general otorhinolaryngology — head and neck surgery practices [2]. Additionally, the most accurate placement of the pH probe has not yet been agreed upon. Despite all of these diagnostic tools, the nasopharynx is historically excluded on clinical investigation in patients with LPR while endoscopic subtleties in the epipharyngeal mucosa may have been missed in the few papers that have addressed the postnasal space in their studies [11–13]. These studies did, however, observe areas of the postnasal space to be highly reliable in response to change. We believe isolated nasopharyngitis is an important finding indicating the presence of LPR. Evidence of upper respiratory inflammation such as rhinosinusitis whether it be acute or chronic would suggest a cause other than LPR. The senior author (JEF) has developed an approach in an iterative manner for diagnosis and management during his 30 years of clinical experience. This evolving method combines a comprehensive history with findings of any degree of nasopharyngitis and/or posterior commissure oedema for a presumptive diagnosis of LPR. The confirmation of the diagnosis is an improvement in symptoms and a reduction or resolution of inflammatory findings following a 3-month course of twice daily low-dose PPI, tapering to a single evening dose for 1 month. This empiric treatment protocol has been adopted by some authors and will be used for the presumptive diagnosis of LPR in this study [14–16]. The aim of our study is to investigate if a direct association between LPR and SRL exists, suggesting LPR is a causative factor in the aetiology of SRL in our patients. We hope to present arguments supporting this conclusion.

## Materials and methods

### Ethical consideration and study design

The Research Ethics Committee of the University Hospital Limerick granted formal approval (Ref 148/2020). A retrospective chart assessment was carried out involving consecutive patient’s that presented to the routine outpatient’s service of the senior author.

### Study population

The study was conducted in an academic tertiary care medical centre. Inclusion criteria included all patients with a history of nocturnal choking attacks. Differential diagnoses such as OSA, nocturnal asthma, sleep terror episodes and seizures were deemed unlikely through careful history and examination. Patients with a history or clinical findings suggestive of any of these differentials were excluded from the study. Patients with suspected OSA or nocturnal asthma were referred to respiratory for further investigations. Patients with a history of nocturnal choking attacks suggestive of SRL subsequently underwent flexible nasopharyngoscopy.

### Methods

Patient charts during a 5-year period from 2016 to 2020 inclusive were examined for patients that presented with or were found on routine history-taking to have symptoms suggestive of SRL. Reflux risk factors for reflux were also recorded. These included smoking status, alcohol intake, a history of dyspepsia or history of gastroesophageal reflux disease, a history of late-night eating and a history of eating spicy or fatty foods before bed. Patients were classified as having risk factors for LPR if any of these criteria were present. Episodes of nocturnal choking were recorded as a presenting symptom or as discovered on direct questioning by including the enquiry as part of a standard history in patients with suspected LPR or sleep disordered breathing.

All patients underwent nasopharyngoscopy by a single consultant otolaryngologist. The presence of nasopharyngitis on endoscopy was noted. Other abnormalities associated with reflux including posterior commissure oedema and hypertrophy were also noted. Criteria for severity of nasopharyngitis are defined in Table 1. Patients with risk factors for LPR or signs of nasopharyngitis on endoscopy were classified as having suspected LPR.

**Table 1:** Nasopharyngitis severity

**Severity**	**Characteristic**
Mild	Minimal erythema with subtle line of demarcation [11, 12]
Moderate	Granular or raised mucosal erythematous changes
Severe	Crusting of the mucosa or blood-stained mucosa

All patients with risk factors for LPR were advised to refrain from eating less than 2 h before bed and to avoid the eating of spicy or fatty foods. Smoking cessation and a reduction in alcohol intake were also recommended if applicable to all patients. Patients without nasopharyngitis on nasendoscopy were advised to take a trial of Gaviscon for 6–8 weeks in addition to lifestyle changes. Patients with signs of nasopharyngitis on nasendoscopy were initiated on a 20 mg twice daily proton pump inhibitor (PPI) for 3 months which was taken 30 min to 1 h before meals. The evening dose was prescribed for a month to prevent rebound symptoms with omission of the morning dose after the initial month. Gavison at night and lifestyle changes were also recommended.

Patients were asked to record any further symptoms or reoccurrences of their nocturnal choking attacks. All patients were monitored remotely by telephone if symptoms failed to settle. Patients with signs of nasopharyngitis on endoscopy were followed up at 4 months with repeat nasopharyngoscopy following PPI therapy.

### Statistical analysis

All statistical analyses were performed using the Statistical Package for the Social Science (SPSS) version 25 software (SPSS Inc., Chicago IL).

## Results and analysis

### Patient demographics

Nineteen patients (mean age ± SD: 57.21 ± 15.18) were included in the study. Fifty-eight percent were female, 10.5% were current smokers and 100% admitted to alcohol intake. None of our patients had a history of epilepsy or seizures and none had a history of anxiety or panic attacks.

### Risk factors for LPR and nasopharyngitis on nasendoscopy

Thirteen patients (68.4%) had a history of dyspepsia or history of gastroesophageal reflux disease. Seven patients (36.8%) had a history of late-night meals or admitted to the eating of spicy or fatty foods less than 2 h before bed. All 19 patients had at least one risk factor for LPR. Eight patients (42.1%) had two risk factors for LPR, and 7 patients (36.8%) had three risk factors. Ten patients (52.6%) had signs of nasopharyngitis on nasendoscopy. There was no correlation between number of risk factors and flexible nasopharyngoscopy findings.

### Posterior commissure hypertrophy/oedema

Seven patients (36.8%) had signs of posterior commissure hypertrophy or oedema on nasendoscopy. Two of 7 (28.6%) patients with posterior commissure hypertrophy did not have signs of nasopharyngitis on nasendoscopy.

### Presenting complaint

Thirteen of the 19 patients presented with nocturnal choking attacks. Number of choking attacks ranged from 1 to 4 attacks per year. Choking attacks lasted less than 10 s in all instances based on patient history and collateral history from partner if available. Six patients (31.5%) did not volunteer episodes at presentation but admitted to nocturnal choking attacks after direct questioning during history taking.

### Symptom reoccurrence

Two of 19 patients (10.5%) had a reoccurrence of symptoms within 1 year of initial consultation. Both patients suffered just one attack each while confirming that a large late meal with excessive alcohol intake preceded the event.

## Discussion

SRL is a condition that has been under-reported in the medical literature. It remains a diagnosis that needs to be considered and studied further to realise its true prevalence and aetiology. This study reveals that SRL is generally not a common problem but is likely to be more frequent than suspected heretofore. It appears to be an infrequent but frightening occurrence for those who have experienced it, although a previously reported series dealt with a group of patients that were more severely impacted including daytime events than any in our cohort [14].

Sudden awakening and nocturnal breathing disturbances can occur for reasons other than SRL. OSA is the most common breathing-related sleep disorder. OSA is characterised by pharyngeal narrowing which results in upper airway obstruction during sleep. These patients typically will not awaken with persistent difficulty breathing or stridor. They also are unlikely to awaken themselves during sleep. OSA patients will also usually have a history of loud snoring and daytime sleepiness. These features are not characteristic of SRL [14]. Patients with suspected OSA were excluded from the study and were referred for sleep studies. Nocturnal asthma is another possible cause of sudden awakening and difficulty breathing. In addition to night-time symptoms, patients with nocturnal asthma will have clinical features of asthma during the day. Symptoms such as persistent dry cough and chest tightness during the day are not typical of SRL. This information was used to exclude patients with suspected nocturnal asthma from the study. Additionally, patients with a pre-existing diagnosis of asthma were excluded from the study. It was anticipated that patients who did not respond to our management regime would require referral to the respiratory team for sleep studies or for assessment of nocturnal asthma. Patients suffering from stress or emotional anxiety can also present with features resembling SRL. Sleep terror episodes can be characterised by sudden arousal accompanied by shortness of breath and behavioural manifestations of intense fear [3]. Seizures may also cause a presentation that is like that of SRL. For this reason, they should be investigated further if suspected. It is important to distinguish between these differential diagnoses when considering SRL as a possible cause of sudden awakening with breathing disturbances. A correct diagnosis of SRL is needed to adequately treat the patient. A detailed history is required from the patient to ensure other differentials are not the cause of the patient’s symptoms. A neurological and otolaryngologic examination should also be performed to identify the aetiology of SRL. All of our patients were careful screened for differential diagnoses and were only included in the study if differentials were ruled out. None of the patients included in the study had proven or suspected OSA, nocturnal asthma, sleep terrors or seizures based on the history and clinical findings.

This study shows a probable association between LPR and SRL. Acute or chronic LPR is likely to be present in all our patients with suspected SRL. We have identified two potential groups. One cohort has no history suggestive of LPR with no evidence of upper aerodigestive tract inflammation and their reflux event is entirely related to a late meal while the second set have ongoing LPR with an exacerbation due to eating later than recommended. All patients had at least one risk factor for LPR while patients with two or three risk factors represented 42% and 37%, respectively. These risk factors can bring about acute episodes of LPR. We hypothesise that acute LPR is the cause of the SRL symptoms seen in our patients who did not have any signs of nasopharyngitis on endoscopy. We believe the management of SRL in these patients should include the eating of food no less than 2 h before bed and to avoid the ingestion of spicy or fatty foods. Alcohol consumption should also be limited and smoking ideally should be ceased.

Nasopharyngitis or epipharyngitis has become more obvious with the advent of the nasopharyngoscope and for those clinicians who specifically look for it during endoscopy. It can be a subtle finding that is easily missed but the line of demarcation as described by Neri et al. does appear to be a clinical indicator of note [12]. The results in our series also show that 53% of patients had signs of nasopharyngitis on nasendoscopy. Nasopharyngitis is believed to occur following exposure of the nasopharynx to chronic LPR. We agree with a recent work that suggested a nasopharyngeal probe be used to assess duration and frequency of reflux events in future studies [10]. In patients with signs of nasopharyngitis on nasendoscopy, we suggest the introduction of a twice daily PPI in addition to the changing of eating habits, Gaviscon prior to bed if there has been a late meal, a limit to alcohol intake and the cessation of smoking. A 3-month course of twice daily low-dose PPI tapering to a single evening dose for 1 month is recommended as per previous authors [15–18].

At 1-year follow-up, 17 patients (89.5%) reported no further SRL symptoms. The two male patients that reported symptoms, did so at Christmas time following a large late meal and the ingestion of excessive amounts of alcohol. Both patients reported only one reoccurrence of symptoms. This suggests that our treatment approach has helped to reduce the frequency of the events in our cohort. It is noteworthy that the majority of our SRL patients were female and presented between the ages of 50 and 70. This is consistent with previous studies on SRL and LPR but contradicts the original paper [2, 14, 19]. This may be due to the relatively rare and fleeting events reported by our patients and that females do tend to seek a medical opinion earlier than males [20, 21]. It may also reflect the mistaken opinion that the event was part of a gasp in patients with sleep apnoea.

The reflux finding score (RFS), published by Belafsky et al. in 2002, considers the presence of posterior commissure hypertrophy in the diagnosis or LPR. Posterior commissure hypertrophy was visualised in seven of our patients. However, the RFS was not used in this study as it does not include the nasopharynx in the diagnosis of LPR [22]. Neither was the reflux symptom index (RSI) used in this study as Chen et al. concluded that no linear relationship existed between RFS and RSI, while they also found asymptomatic patients could present with relatively high RFS scores [23]. Although coughing after eating or after lying down and breathing difficulties or choking episodes are included as two of the nine symptoms in the RSI, patients with LPR are considered usually upright refluxers and SRL has not been described in this outcome instrument [22]. Neither has SRL been alluded to in other more recently published scoring systems or LPR reviews [7–10].

We feel that the nasopharynx should be scrutinised more carefully and that this could have altered the significance of nasopharyngitis and the RSA score in the study by Lechien et al. [13]. In an era where patient reported outcome measures (PROMs) are topical, the ability to listen to the patient and clinical experience are paramount. We would like to highlight the importance of history taking in the diagnosis of SRL. A proportion of our population (31.5%) did not volunteer symptoms of SRL and only admitted to nocturnal choking attacks after direct questioning. SRL is a condition that appears to occur sporadically and can be easily overlooked by patients and physicians during history taking. It is our belief that SRL is largely under-diagnosed for this reason. It is our recommendation that all patients with a suspected history of LPR or sleep-disordered breathing should be screened for SRL by asking about nocturnal choking attacks. Attention to detail in listening to the patient will also help to differentiate the episodes of SRL from the more common sleep disturbance in sleep apnoea.

Although there is no evidence-base for SRL nor for nasopharyngitis as a sign of LPR, patients with a worrying complaint were managed successfully with relatively easy and straightforward solutions. Limitations to this study include its small sample size and the lack of a definitive diagnostic test. Currently, the diagnosis depends on a combination of factors including the description of symptoms, the elimination of other potential causes, the presence or absence or nasopharyngitis, and the response to empiric management. Due to the prevalence of SRL, a small sample size is an issue that is hard to overcome in a single tertiary care medical centre. We would suggest future studies include multiple sites for a better representation of the population. Additionally, future studies would ideally include the use of overnight polysomnogram and pH monitoring as has been demonstrated in previous studies [2]. However, our recommendation would be, if possible, to place the upper pH probe in the nasopharynx coupling with a hypopharyngeal sensor. Despite the limitations, this study produced results suggesting that a relationship between SRL and LPR exists. The extent of this relationship requires additional investigation and the inclusion of a control group. Future studies should focus on increasing the study population to further investigate the treatment of SRL through the management of LPR. The response of nasopharyngitis to empiric PPI treatment for LPR is currently the subject of an ongoing retrospective study at our department.

## Conclusion

SRL is a largely unknown and under-diagnosed condition. Additional research into the true aetiology of SRL is required to successfully treat the disorder. We believe this study provides supportive evidence for the causal relationship between LPR and SRL. As such, it would suggest that SRL can and should be treated by limiting the risk of LPR. We also recommend the screening of all patients with a history of reflux for SRL as symptoms are often not volunteered during the history.
